# A Literary Evening at the Watershed Centre

**Published:** 1982

**Authors:** Oliver Neville, Pat Heywood


### A Literary Evening at the Watershed Centre

**Oliver Neville and Pat Heywood**

The final meeting of the 1981–82 session of the Bristol Medico-Chirurgical Society at the newly opened Watershed Centre. We have enjoyed an excellent buffet supper, the wine was good and plentiful, we have had coffee, we repair to the theatre, the seats are unusually comfortable. The lights go down, on stage a table piled with books, seated behind them are *Dr. Oliver Neville,* University Department of Drama and his wife *Pat Heywood,* the actress. They take it in turns to read to us.

1. Pat Heywood – on *Dr. Isaac Letsome.* Anon.
*When people's ill they comes to I,*
*I physics, bleeds and sweats em',*
*Sometimes they lives, sometimes they dies;*
*But what's that to I? I letsome!*

2. Oliver Neville – When Dr. Warin invited us to present a group of readings, related to Medicine, to your Society, Pat and I were both delighted to be asked and also intrigued – for as it happens, we have a number of Doctors of various kinds and ages within our family circle. In making our programme we first looked at laymen writing about doctors and found that they were extremely critical (Dickens, Flaubert, Fielding, Sterne and Moliere) – and we thought that that kind of talk was the last thing you would wish after your annual dinner. So we turned to Doctors writing about Doctors (Rabelais, Bridie, Conan Doyle, Maugham and Chekov): and found that they were even more critical than the laymen – in some cases positively vindictive. So we've chosen a mixture – of laymen on doctors, and doctors on doctors. And have interspersed our readings with anecdotes from a seemingly endless supply of witty practitioners. Pat has already given you one example. To begin on a reasonably positive note, we have a passage from *George Eliot's Middlemarch* (1871) – which describes how the young surgeon/physician Dr. Lydgate, came to a sense of his vocation.

3. Oliver Neville, *Middlemarch,* chapter 15. 'He had been left an orphan when he was fresh from a public school. His father, a military man, had made but little provision for three children and when the boy Tertius asked to have a medical education, it seemed easier to his guardians to grant his request by apprenticing him to a country practitioner than to make any objections on the score of family dignity. He was one of the rarer lads who early get a decided bent and make up their minds that there is something in life which they would like to do for its own sake, and not because their fathers did it. Most of us who turn to any subject we love remember some morning or evening hour when we got on a high stool to reach down an untried volume, or sat with parted lips listening to a new talker, or for very lack of books began to listen to the voices within, as the first traceable beginning of our love. Something of that sort happened to Lydgate. He was a quick fellow, and when hot from play, would throw himself into a corner, and in five minutes be deep in any sort of book that he could lay his hands on: if it were Rasselas or Gulliver, so much the better, but Bailey's Dictionary would do, or the Bible with the Apocrypha in it. Something he must read if he were not riding the pony, or running and hunting, or listening to the talk of men. All this was true of him at ten years of age. He was a vigorous animal with a ready understanding, but no spark had yet kindled in him an intellectual passion; knowledge seemed to him a very superficial affair, easily mastered: judging from the conversation of his elders, he had already got more than was necessary for mature life. But, one vacation, a wet day sent him to the small home library to hunt once more for a book which might have some freshness for him: in vain! unless, indeed, he took down a dusty row of volumes with grey paper backs and dingy labels – the volumes of an old Cyclopoedia which he had never disturbed. It would at least be a novelty to disturb them. They were on the highest shelf, and he stood on a chair to get them down. But he opened the volume which he first took from the shelf: somehow, one is apt to read in a makeshift attitude, just where it might seem inconvenient to do so. The page he opened on was under the heading of Anatomy, and the first passage which drew his eyes was on the valves of the heart. He was not much acquainted with valves of any sort, but he knew that valvae were folding doors, and through this crevice came a sudden light startling him with his first vivid notion of finely adjusted mechanism in the human frame. A liberal education had of course left him free to read the indecent passages in the school classics, but beyond a general sense of secrecy and obscenity in connection with his internal structure, had left his imagination quite unbiased, so that for anything he knew his brains lay in small bags at his temples, and he had no more thought of representing to himself how his blood circulated than how paper served instead of gold. But the moment of vocation had come, and before he got down from his chair, the world was made new to him by a presentiment of endless processes filling the vast spaces planked out of his sight by that wordy ignorance which he had supposed to be knowledge. From that hour Lydgate felt the growth of an intellectual passion.'

4. Pat Heywood – *Martin Chuzzlewit, Charles Dickens.* Ch. 25. "Is in part professional; and furnishes the reader with some valuable hints in relation to the management of a sick chamber."

Regarding herself as having now delivered her inaugural address, Mrs. Gamp curtsied all round, and signified her wish to be conducted to the scene of her official duties. The chambermaid led her, through a variety of intricate passages to the top of the house; and pointing at length to a solitary door at the end of a gallery, informed her that yonder was the chamber where the patient lay.

Mrs. Gamp traversed the gallery in a great heat from having carried her large bundle up so many stairs, and tapped at the door, which was immediately opened by Mrs. Prig, bonnetted and shawled and all impatience to be gone. Mrs. Prig was of the Gamp build but not so fat. 'I began to think you warn't a-coming! Mrs. Prig observed, in some displeasure.

'It shall be made good tomorrow night,' said Mrs. Gamp, 'honourable, I had to go and fetch my things'. She had begun to make signs of enquiry in reference to the position of the patient and his overhearing them – for there was a screen before the door – when Mrs. Prig settled that point easily.

'Oh!' she said aloud, 'he's quiet, but his wits is gone. It an't no matter wot you say.' 'Anythin' to tell afore you goes my dear?' asked Mrs. Gamp, setting her bundle down inside the door, and looking affectionately at her partner.

'The pickled salmon,' Mrs. Prig replied, 'is quite delicious. I can partick'ler recommend it. Don't have nothink to say to the cold meat, for it tastes of the stable. The drinks is all good.'

Mrs. Gamp expressed herself much gratified.

'The physic and them things is on the drawers and mankleshelf' said Mrs. Prig cursorily. 'He took his last slime draught at seven. The easy chair an't soft enough. You'll want his piller.'

Mrs. Gamp thanked her for these hints, and giving her a friendly good night, held the door open until she had disappeared from the other end of the gallery. Having thus performed the hospitable duty of seeing her safely off, she shut it and locked it on the inside, took up her bundle, walked round the screen, and entered on her occupation of the sick chamber.

'A little dull, but not so bad as might be,' Mrs. Gamp remarked. 'I'm glad to see a parapidge, in case of fire, and lots of roofs and chimley pots to walk upon.'

It will be seen from these remarks that Mrs. Gamp was looking out of the window. When she had exhausted the prospect, she tried the easy chair, which she indignantly declared was 'harder than a brickbadge'. Next she pursued her researches among the physic bottles, glasses, jugs and teacups: and when she had entirely satisfied her curiosity on all these subjects of investigation, she untied her bonnet strings and strolled up to the bedside to take a look at the patient.

A young man – dark and not ill-looking – with long black hair, that seemed the blacker for the whiteness of the bedclothes. His eyes were partly open, and he never ceased to roll his head from side to side upon the pillow, keeping his body almost quiet. He did not utter words; but every now and then gave vent to an expression of impatience or fatigue, sometimes of surprise; and still his head – oh, weary, weary hour! – went to and fro without a moment's intermission.

Mrs. Gamp solaced herself with a pinch of snuff, and stood looking at him with her head inclined a little sideways, as a connoisseur might gaze upon a doubtful work of art. By degrees a horrible remembrance of one branch of her calling took possession of the woman; and stooping down, she pinned his wandering arms against his sides, to see how he would look if laid out as a dead man. Hideous as it may appear, her fingers itched to compose his limbs in that last marble attitude.

'Ah!' said Mrs. Gamp, walking away from the bed, 'he'd make a lovely corpse.'

She now proceeded to unpack her bundle; lighted a candle with the aid of a firebox on the drawers; filled a small kettle, as a preliminary to refreshing herself with a cup of tea in the course of the night; laid what she called a 'little bit of fire', for the same philanthropic purpose; and also set forth a small teaboard, that nothing might be wanting for her comfortable enjoyment. These preparations occupied so long, that when they were brought to a conclusion it was high time to think about supper, so she rang the bell and ordered it.

'I think, young woman,' said Mrs. Gamp to the assistant chambermaid, in a tone of expressive weakness, 'that I could pick a bit of pickled salmon, with a nice little sprig of fennel, and a sprinkling of white pepper. I takes new bread, my dear, with just a little pat of fresh butter, and a mossel of cheese. In case there should be such a thing as a cowcumber in the 'ouse, will you be so kind as to bring it, for I'm rather partial to 'em, and they does a world of good in a sick room. If they draws the Brighton Old Tipper here, I takes that ale at night my love; it bein' considered wakeful by the doctors. And whatever you do young woman, don't bring more than a shillings-worth of gin and water-warm when I rings the bell a second time; for that is always my allowance, and I never takes a drop beyond!'

Having preferred these moderate requests. Mrs. Gamp observed that she would stand at the door until the order was executed, to the end that the patient might not be disturbed by her opening it a second time; and therefore she would thank the young woman to 'look sharp'.

A tray was brought with everything upon it, even to the cucumber; and Mrs. Gamp accordingly sat down to eat and drink in high good humour. The extent to which she availed herself of the vinegar and supped up that refreshing fluid with the blade of her knife, can scarcely be expressed in narrative.

'Ah!' sighed Mrs. Gamp, as she meditated over the warm shillings-worth, 'what a blessed thing it is – living in a wale – to be contented! What a blessed thing it is to make sick people happy in their beds, and never mind one's self as long as one can do a service! I don't believe a finer cowcumber was ever growed. I'm sure I never see'd one'!

She moralised in the same vein until her glass was empty, and then administered the patient's medicine, by the simple process of clutching his windpipe, to make him gasp, and immediately pouring it down his throat.

'I a'most forgot the piller, I declare' said Mrs. Gamp, drawing it away. 'There! Now he's as comfortable as can be, I'm sure I must try to make myself as much so as I can.'

With this view, she went about the construction of an extemporaneous bed in the easy-chair, with the addition of the next easy one for her feet. Having formed the best couch that the circumstances permitted of, she took out of her bundle a yellow night-cap, of prodigious size, in shape resembling a cabbage; which article of dress she fixed and tied on with the utmost care, previously divesting herself of a row of bald old curls that could scarcely be called false, they were so very innocent of anything approaching to deception. From the same repository she brought forth a night-jacket, in which she also attired herself. Finally, she produced a watchman's coat, which she tied round the neck by the sleeves, so that she became two people; and looked, behind as if she were in the act of being embraced by one of the old patrol.

All these arrangements made, she lighted the rush light, coiled herself up on her couch, and went to sleep.

5. Oliver Neville – *On the Death of Dr. Robert Levet, Samuel Johnson.* It is well known that Samuel Johnson, the great lexicographer, lived in London with a cat named Hodge, who was very partial to oysters. Among Dr. Johnson's other indigent tenants was an obscure, brusque doctor named Levet – who devoted himself to the care of the poor. Johnson had known Levet upwards of some 30 years, and later sheltered him until his death on 17th January, 1782. Johnson wrote the following poem: restrained but informed with the sincerity of deep personal grief.

*Condemned to hope's delusive mine,*
*As on we toil from day to day,*
*By sudden blasts or slow decline,*
*Our social comforts drop away.*

*Well tried through many a varying year,*
*See Levet to the grave descend,*
*Officious, innocent, sincere,*
*Of every friendless name the friend.*

*Yet still he fills affection's eye,*
*Obscurely wise and coarsely kind;*
*Nor, lettered arrogance deny*
*Thy praise to merit unrefined.*

*When fainting nature call'd for aid,*
*And hov'ring death prepared the blow,*
*His vig'rous remedy displayed*
*The power of art without the show.*

*In misery's darkest caverns known,*
*His useful care was ever nigh,*
*Where hopeless anguish poured his groan,*
*And lonely want retired to die.*

*No summons mocked by chill delay,*
*No petty gain disdained by pride,*
*The modest wants of every day*
*The toil of every day supplied.*

*His virtues walked their narrow round,*
*Nor made a pause, nor left a void;*
*And sure th' Eternal Master found*
*The single talent well employed.*

*The busy day, the peaceful night,*
*Unfelt, uncounted, glided by;*
*His frame was firm, his powers were bright,*
*Tho' now his eightieth year was nigh.*

*Then with no throbbing fiery pain,*
*No cold gradations of decay,*
*Death broke at once the vital chain,*
*And freed his soul the nearest way.*

6. Pat Heywood – *On Plastic Surgery, Sir Archibald McIndoe.*

*I do not like surgeons,*
*They are a brutal race,*
*They came and sliced my tummy up*
*And parked it on my face.*

*I do not like surgeons,*
*For they are not refined.*
*Who wants to have a forehead made*
*Of bits from her behind?*

7. Oliver Neville – Sir Arthur Conan Doyle took his medical degree in Edinburgh: and practiced for eight years – being Senior Physician to a field hospital in the South African War. *'Behind the times"* from *Tales of Adventure and Medical Life Conan Doyle* (1922).

My first interview with Dr. James Winter was under dramatic circumstances. It occurred at two in the morning in the bedroom of an old country house. I kicked him twice in the white waistcoat and knocked off his gold spectacles, while he with the aid of a female accomplice, stifled my cries in a flannel petticoat and thrust me into a warm bath. I am told that one of my parents, who happened to be present, remarked in a whisper that there was nothing the matter with my lungs. I cannot recall how Dr. Winter looked at the time, for I had other things to think of, but his description of my appearance is far from flattering. A fluffy head, a body like trussed goose, very bandy legs, and feet with the soles turned inwards – those are the main items which he can remember.

From this time onwards the epochs of my life were the periodic assaults Dr. Winter made upon me. He vaccinated me, he cut me for an abscess, he blistered me for mumps. It was a world of peace, and he the one dark cloud that threatened. But at last there came a time of real illness – a time when I lay for months together inside my wickerwork basket bed, and then it was that I learned that that hard face could relax, that those country-made, creaking boots could steal very gently to a bedside, and that that rough voice could thin into a whisper when it spoke to a sick child.

And now the child himself is a medical man, and yet Dr. Winter is the same as ever. I can seen no change since first I can remember him, save that perhaps the brindled hair is a trifle whiter, and the huge shoulders a little more bowed. he is a very tall man though he loses a couple of inches from his stoop. That big back of his has curved itself over sick beds until it has set itself in that shape. His face is of a walnut brown, and tells of long winter drives over bleak country roads with the wind and the rain in his teeth. It looks smooth at a little distance, but as you approach you see that it is shot with innumerable fine wrinkles, like a last year's apple. They are hardly to be seen when it is in repose, but when he laughs his face breaks like a starred glass, and you realise then that, though he looks old, he must be older than he looks.

But it was only when I had myself become a medical man that I was able to appreciate, how entirely he is a survival of a past generation. He had learned his medicine under that obsolete and forgotten system by which a youth was apprenticed to a surgeon, in the days when the study of anatomy was often approached through a violated grave. Fifty years have brought him little and deprived him of less. Vaccination was well within the teaching of his youth, though I think he has a preference for innoculation. Bleeding he would practice freely were it not for public opinion. Chloroform he regards as a dangerous innovation, and he always clicks his tongue when it is mentioned. He has even been known to refer to the stethoscope as a 'new-fangled French toy'. He carried one in his hat out of deference to the expectations of his patients; but he is very hard of hearing so that it makes little difference whether he uses it or not.

He always reads as a duty his weekly medical paper, so that he has a general idea as to the advance of modern science. He persists in looking upon it however as a huge and rather ludicrous experiment. He is so very much behind the day that occasionally, as things move round in their usual circle, he finds himself, to his own bewilderment, in the front of the fashion. Dietetic treatment, for example, had been much in vogue in his youth, and he has a more practical knowledge of it than anyone whom I have met. Massage too, was familiar to him when it was new to our generation. He had been trained also at a time when instruments were in a rudimentary state and when men learned to trust more in their own fingers. He has a model surgical hand, muscular in the palm, tapering in the fingers, 'with an eye at the end of each'. I shall not easily forget how Dr. Patterson and I cut Sir John Sirwell, the County member, and were unable to find the stone. It was a horrible moment. Both our careers were at stake. And then it was that Dr. Winter, whom we had asked out of courtesy to be present, introduced into the wound a finger which seemed to our excited senses to be about nine inches long, and hooked the stone out at the end of it.

'It's always well to bring one in your waistcoat pocket' said he with a chuckle, 'but I suppose you youngsters are above all that'.

We made him President of our branch of the British Medical Association, but he resigned after the first meeting. 'The young men are too much for me' he said. 'I don't understand what they are talking about'. Yet his patients do very well. He has the healing touch – that magnetic thing which defies explanation or analysis, but which is a very evident fact none the less. His mere presence leaves the patient with more hopefulness and vitality. The sight of disease affects him as dust does a housewife. It makes him angry and impatient. 'Tut, tut, this will never do' he cries, as he takes over a new case. He would shoo Death out of the room as though he were an intrusive hen. But when the intruder refuses to be dislodged, when the blood moves more slowly and the eye grows dimmer, then it is that Dr. Winter is of more avail than all the drugs in his surgery. Dying folk cling to his hand as if the presence of his bulk and vigour gives them more courage to face the change; and that kindly and wind beaten face has been the last earthly impression which many a sufferer has carried into the unknown.

When Dr Patterson and I, both of us young, energetic and up-to-date, settled in the district, we were most cordially received by the old doctor, who would have been only too happy to have been relieved of some of his patients. The patients themselves, however followed their own inclinations, which is a reprehensible way that patients have, so that we remained neglected with our modern instruments and our latest alkaloids, while he was serving out senna and calomel to all the countryside. We both of us loved the old fellow, but at the same time, in the privacy of our own intimate conversations, we could not help commenting on this deplorable lack of judgement.

'It is all very well for the poorer people', said Patterson, 'but after all the educated classes have a right to expect that their medical man will know the difference between a mitral murmer and a bronchitic rale. It's the judicial frame of mind, not the sympathetic, which is the essential one.'

I thoroughly agreed with Patterson in what he said. It happened, however, that very shortly afterwards the epidemic of influenza broke out, and we were all worked to death. One morning I met Paterson on my round, and found him looking rather pale and fagged out. He made the same remark about me. I was in fact feeling far from well, and I lay upon the sofa all afternoon with a splitting headache and pains in every joint. As evening closed in I could no longer disguise the fact that the scourge was upon me, and I felt that I should have medical advice without delay. It was of Patterson naturally that I thought, but somehow the idea of him had suddenly become repugnant to me. I thought of his cold critical attitude, of his endless questions, of his tests and his tappings. I wanted something more soothing, something more genial.

'Mrs. Hudson' said I to my housekeeper. 'Would you kindly run along to old Dr. Winter and tell him that I should be obliged to him if he would step round'.

She came back with an answer presently.

'Dr. Winter will come round in a hour or so, sir, but he has just been called in to attend to Dr. Patterson'.

8. Oliver Neville and Pat Heywood – *Edward Jenner* sent the following epigram with a present of a couple of ducks to a patient –

*I've dispatched, my dear madam, this scrap of a letter*
*To say that Miss X is very much better.*
*A regular doctor no longer she lacks,*
*And therefore I've sent her a couple of quacks.*

To which came the following reply –

*Yes! 'Twas politic truly, my very good friend,*
*Thus a couple of quacks to your patient to send;*
*Since there's nothing so likely as quacks (it is plain)*
*To make work for a regular doctor again.*

9. Pat Heywood – *Madam Bovary (1857) by Gustave Flaubert.*

[Charles Bovary is a country practitioner. He has just discovered that owing to the extravagance of his wife Emma, he is financially ruined. He returns home to confront her – unaware that sometime previously she has taken arsenic.]

When Charles got home, overwrought at the news of the distraint, Emma had just gone. He called for her, wept, but she did not return. Where could she be? He sent Félicité to the Homais', to Monsieur Tuvache's, to Lheureux's, to the Golden Lion, everywhere; and in the intervals of his anguish he saw his reputation lost, his money gone, his daughter's future wrecked. Through what cause?

No word to tell him. He waited till six o'clock, then he could stand it no longer. Fancying that she must have set off for Rouen, he walked a mile and a half along a highroad, met nobody, waited a while, turned back home again.

She had come in.

'What happened? What was it? Tell me.'

She sat down at her desk and wrote a letter, which she slowly sealed, adding the date and time.

'Read it tomorrow' she said in a solemn voice. 'Till then please don't ask me a single question. . . . No, not one.'

'But. . . .'

'No, leave me alone.'

She lay down full length on the bed.

An acrid taste in her mouth awoke her. She saw Charles and closed her eyes again. She watched with curiosity for any sign of pain. No, nothing yet. She heard the clock ticking, the fire crackling, Charles breathing as he stood beside her.

'There's not much in dying' she thought. 'I shall go to sleep and it will all be over.'

She gulped down some water, and turned to the wall. That dreadful inky taste was still there. 'I'm thirsty, I'm so thirsty' she whispered.

'What can it be?' said Charles, handing her the glass. It's nothing. . . . Open the window. . . . I feel choked.' And she vomited so suddenly that she barely had time to grab her handkerchief from under the pillow. 'Take it out. Throw it away' she said quickly.

He started questioning her. She didn't answer. She kept quite still, afraid that the least agitation might make her sick. She began to feel an icy coldness mounting from her feet towards her heart. 'That's it, it's beginning,' she mumbled.

'What did you say?'

She rolled her head with a gentle, anguished movement, trying to open her jaws all the while as though she had a heavy weight on her tongue. At eight o'clock the vomiting began again.

Charles noticed a sort of white sediment clinging to the bottom of the basin. 'That's funny, that's queer' he kept saying.

'No, it's nothing' she said in a firm voice.

Very gently, almost caressingly, he passed his hand over her stomach. She gave a shriek, he started back in terror.

She began to groan, feebly at first. A violent shudder went through her shoulders, she turned whiter than the sheet she was clutching in her fingers. Her wavering pulse could hardly be felt at all now.

Drops of sweat stood on her pale blue-veined face, which looked as if it had been petrified by exposure to some metallic vapour. Her teeth chattered, her pupils were dilated, her eyes stared vaguely about her. To every question she responded simply with a shake of the head. Two or three times she smiled. Little by little her groans grew louder. A muffled scream broke from her. She pretended to be feeling better and said she would soon be getting up. But then she was seized with convulsions. 'Oh, God, it's ghastly' she cried.

He went down on his knees beside her. 'Tell me what you've eaten. For God's sake answer'. And he looked at her with such tenderness in his eyes as she had never seen before.

'All right,' she said in a faltering voice. 'There, over there. . . .'

He sprang to the desk, broke the seal and read aloud, 'No one is guilty . . .' he stopped, passed his hand over his eyes, read on.

'What. . . . Help, Oh help.' And he could only utter the one word, 'Poisoned, poisoned!'

Félicité ran to get Homais, who shouted the news across the square. Madam Lefrancois heard him at the Golden Lion, several people got out of bed to tell their neighbours, and the village was awake all night.

He went over to her again, sank down on the carpet, and knelt with his head against the edge of the bed sobbing.

'Don't cry' she said. 'Soon I shall be troubling you no more.'

She had done, she was thinking, with all the treachery and the squalor and the numberless desires that had racked her. She hated no one now; a twilight confusion was descending on her mind.

She seemed less agitated, and at every slightest word, at every quieter breath she drew, Charles took fresh hope. When Canivet at last walked in Charles threw himself into his arms, weeping.

'It's you. Thank you, it is kind of you. But she's better now. Look!'

His colleague by no means shared this opinion; and without – as he put it – beating about the bush, he prescribed an emetic to clear the stomach completely.

Not long afterwards she started vomiting blood. Her lips pressed together tighter, her limbs writhed, brown spots broke out over her body, her pulse slid between the fingers like a taut wire, like a harp string about to snap.

She began to scream horribly. She damned and cursed the poison, begging it to be quick, and with her stiffening arms pushed away everything that Charles, in a still worse agony than she, kept trying to make her drink. He stood by with his handkerchief to his lips, croaking and crying, choked by the sobs that shook his frame.

And just then she looked all around her slowly, as one waking from a dream. In a clear voice she asked for her mirror, and remained bowed over it for some time, until big tears began to trickle out of her eyes. Then she threw up her head with a sigh and fell back on to the pillow.

At once her lungs began to heave rapidly, the whole of her tongue protruded from her mouth, her rolling eyes turned pale like the globes of two guttering lamps: she might have been dead already but for the frightful oscillation of her ribs, that shook with furious gusts as though her soul were leaping to get free.

A convulsion flung her down upon the mattress. They moved nearer. She was no more.

10. Oliver Neville – *Laurence Sterne* wrote *Tristram Shandy* in the 1750's.

[The characters – Tristram's father and his Uncle Toby. Corporal Trim, servant to Uncle Toby and his one-time Army Batman. Obadiah and Susannah are servants, and our Medical Practitioner is Dr. Slop. Tristram's father and Uncle Toby sit smoking in the back parlour of Shandy Hall, mulling over the problems of Mrs. Shandy, who is upstairs in bed, awaiting the imminent arrival of Baby Tristram. The story is told in retrospect by Tristram himself.]

'My father, who dipped into all kinds of books, had found out that the lax and pliable state of a child's head in parturition, the bones of the cranium having no sutures at that time, was such – that by force of the woman's efforts, which, in strong labour pains, was equal, upon an average, to a weight of 470 lbs. averdupoise acting perpendicularly upon it – it so happened that in 49 instances out of 50, the said head was compressed and moulded in to the shape of an oblong conical piece of dough, such as a pastry-cook generally rolls up in order to make a pie of – Good God! cried my father, what havoc and destruction must this make in the infinitely fine and tender texture of the cerebellum!

But how great was this apprehension when he further understood, that this force, acting upon the very vertex of the head, not only injured the brain itself or cerebrum – but that it necessarily squeezed and propelled the cerebrum towards the cerebellum, which was the immediate seat of the understanding! – Angels and Ministers of grace defend us, cried my father – can any soul withstand this shock? – No wonder the intellectual web is so rent and tattered as we see it; and that so many of our best heads are no better than a puzzled skein of silk, – all perplexity – all confusion within-side.

But when my father read on, and was let in to the secret, that when a child was turned topsy-turvy, which was easy for an operator to do, and was extracted by the feet; – that instead of the cerebrum being propelled towards the cerebellum, the cerebellum, on the contrary, was propelled simply towards the cerebrum where it could do no manner of hurt: – By Heavens cried he, the world is in a conspiracy to drive out what little wit God has given us – and the professors of the obstetric art are listed into the same conspiracy – What is it to me which end of my son comes foremost into the world, provided all goes right after, and his cerebellum escapes uncrushed?

This was my father Mr. Shandy's hypothesis; concerning which I have only to add, that my brother Bobby did as great honour to it – coming into the world with his head foremost – and turning out afterwards a lad of wonderful slow parts – my father spelt all these together into his opinion; and as he had failed at one end – he was determined to try the other. This was therefore one of my father's great reasons in favour of a man of science whom he could better deal with.

Of all the men in the world, Dr. Slop was the fittest for my father's purpose – for, though his new invented forceps was the armour he had proved, and what he maintained, to be the safest instrument of deliverance – yet, it seems, he had scattered a word or two in his book, in favour of the very thing which ran in my father's fancy – though not with a view to the soul's good in extracting by the feet, as was my father's system – but for reasons merely obstetrical. This will account for the coalition betwixt my father and Dr Slop in the following discourse.

What can they be doing brother? said my father – I think replied my Uncle Toby – taking, as I told you, his pipe from his mouth, and striking the ashes out of it as he began his sentence – I think, replied he – it would not be amiss brother, if we rung the bell.

Pray what's all that racket over our heads, Obadiah? – quoth my father – my brother and I can scarce hear ourselves speak.

Sir, answered Obadiah, making a bow towards his left shoulder – my Mistress is taken very badly – and where's Susannah running down the garden there, as if they were going to ravish her? – Sir, she is running the shortest cut into the town, replied Obadiah, to fetch the old midwife. Then saddle a horse, quoth my father, and do you go directly for Dr Slop the man-midwife, with all our services – and let him know that your Mistress is fallen into labour – and that I desire he will return with you with all speed.

It is very strange, says my father, addressing himself to my Uncle Toby, as Obadiah shut the door – as there is so expert an operator as Dr. Slop so near – that my wife should persist to the very last in this obstinate humour of hers, in trusting the life of my child, who has had one misfortune already, to the ignorance of an old woman – and not only the life of my child, brother – but her own life, and with it the lives of all the children I might peradventure, have begot out of her herefter.

Mayhap, brother, replied my Uncle Toby, my sister does it to save the expense; – A pudding's end – replied my father – the doctor must be paid the same for inaction as action – if not better – to keep him in temper.

Then it can be out of nothing in the whole world, quoth my Uncle Toby but Modesty – My sister, I dare say, he added, does not care to let a man come so near her ***** I will not say whether my Uncle Toby had completed this sentence or not – 'tis for his advantage to suppose he had – as, I think, he could have added no one word which would have improved it. But whether that was the case or not, or whether the snapping of my father's tobacco pipe so critically, happened through accident or anger – will be seen in due time.

Imagine to yourself a little squat, uncourtly figure of a Doctor Slop, of about four feet and a half perpendicular height, with a breadth of back, and a sesquipedality of belly, which might have done honour to a serjeant in the horse guards.

Imagine such a one – for such, I say, were the outlines of Dr. Slop's figure, coming slowly along, foot by foot, waddling through the dirt upon the vertebrae of a little diminutive pony, of a pretty colour – but of strength – slack – scarce able to have made an amble of it, under such a fardel, had the roads been in an ambling condition. – They were not – Imagine to yourself, Obadiah mounted upon a strong monster of a coach horse, pricked into a full gallop, and making all practicable speed the adverse way.

Dr Slop was advancing thus warily along towards Shandy Hall, and had approached to within sixty yards of it, and within five yards of a sudden turn, made by an acute angle of the garden wall, and in the dirtiest part of the lane – when Obadiah and his coach horse turned the corner, rapid, furious, – pop, – full upon him. – Nothing, I think, in nature, can be supposed more terrible than such a Rencounter – so imprompt, so ill prepared to stand the shock of it as Dr Slop was.

What could Dr Slop do? – He crossed himself – Pugh! – but the doctor, Sir, was a Papist – No matter; he had better have kept hold of the pummel. He had so – nay as it happened, he had better have done nothing at all – for in crossing himself he let go his whip – and in attempt to save his whip betwixt his knee and his saddle's skirt, as it slipped, he lost his stirrup, – and in loosing which, he lost his seat, – and in the multitude of all these losses (which, by the bye, shews what little advantage there is in crossing) the unfortunate doctor lost his presence of mind. So, that, without waiting for Obadiah's onset, he left his pony to its destiny, tumbling off it diagonally, something in the stile and manner of a pack of wool, and without any other consequence from the fall, save that of being left (as it would have been) with the broadest part of him sunk about twelve inches deep in the mire.

When Dr. Slop entered the back parlour, where my father and Uncle Toby were discoursing on the nature of women – it was hard to determine whether Dr. Slop's figure, or Dr. Slop's presence, occasioned more surprise to them; for as the accident happened so near the house as not to make it worth while for Obadiah to remount him – Obadiah had led him in as he was, unwiped, unappointed, unannealed, with all his stains and blotches upon him – He stood like Hamlet's ghost, motionless and speechless, for a full minute and a half, at the parlour door (Obadiah still holding his hand) with all the majesty of mud.

Let the reader imagine then, that Dr Slop has told his tale; – and in what words, and with what aggravation his fancy chooses; – Let his suppose, that Obadiah has told his tale also, with such rueful looks of affected concern, as he thinks will best contrast the two figures as they stand by each other; – Let him imagine, that my father has stepped upstairs to see my mother; – and to conclude this sort of imagination – let him imagine the doctor washed, rubbed down, condoled with, felicitated, got into a pair of Obadiah's pumps, upon the very point of entering action.

For happening to have his green bays bag upon his knees (containing tire-tête forceps, crotchet and squirt: indeed for all his instruments of salvation and deliverance), he thrust his hand into the bag in order to have his newly invented forceps ready to clap in. But Dr. Slop fumbled so vilely in pulling them out, it took off the whole effect, and what was a ten times worse evil (for they seldom come alone in this life) in pulling out his forceps, his forceps unfortunately drew out the squirt along with it.

'Good God', cried my Uncle Toby, 'are children brought in to the world with a squirt?'

The midwife arrived in time to witness Dr. Slop demonstrating his invention, using Uncle Toby's fists for the baby's head.

Upon my honour, Sir, you have torn every bit of the skin off the back of both of my hands with your forceps, cried my Uncle Toby – and you have crushed all my knuckles into the bargain with them to a jelly. 'Tis your own fault' said Dr. Slop – you should have clinched your two fists into the form of a child's head, as I told you, and sat firm. I did so, answered my Uncle Toby.

Then the point of my forceps have not been sufficiently armed, or the rivets want closing, or else the cut on my thumb has made me a little awkward – or possibly – 'Tis well quoth my father interrupting the detail of possibilities – that the experiment was not first made upon my child's head piece.

It would not have been a cherry stone the worse, answered Dr. Slop. I maintain it, said my Uncle Toby, would have broke the cerebellum (unless indeed the skull had been as hard as a granado) and turned it all into a perfect possett. Pshaw! replied Dr. Slop, a child's head is naturally as soft as the pap of an apple – the sutures give way, and besides, I could have extracted it by the feet after.

I rather wish you would begin that way, quoth my father.

'Pray do, added my Uncle Toby.

It is morally impossible the reader should understand this – 'tis enough Dr. Slop understood it. So taking the green bays bag in his hand, with the help of Obadiah's pumps, he tripped pretty nimbly for a man of his size, across the room to the door – and from the door was shown the way by the good midwife, to my mother's appartment.

Two hours and ten minutes later, when Corporal Trim came in and told my father that Dr Slop was in the kitchen, and busy in making a bridge – my Uncle Toby took it instantly for granted that Dr. Slop was repairing his model drawbridge of the siege of Namur, which was broke down and somewhat crushed to pieces that very night.

'Tis very obliging in him, quoth my Uncle Toby – pray give my humble service to Dr. Slop and tell him I thank him heartily.

But Trim's answer, in an instant, tore the laurel from his brows, and twisted it in pieces. God bless your honour, cried Trim, 'tis a bridge for master's nose – In bringing him into the world with his vile instruments, he has crushed his nose, Susannah says, as flat as a pancake to his face, and he is making a false bridge with a piece of cotton and a thin piece of whalebone out of Susannah's stays, to raise it up.

Lead me, brother Toby, cried my father, to my room this instant.

11. Pat Heywood – *Oliver Goldsmith* (won renown as playwright, novelist and poet, Was a failure as a doctor).

From *The Vicar of Wakefield – An elegy on the death of a Mad Dog.*

*An Elegy on the Death of a Mad Dog*

*Good people all, of every sort,*
*Give ear unto my song,*
*And if you find it wondrous short,*
*It cannot hold you long.*

*In Islington there was a man,*
*Of whom the world might say,*
*That still a goodly race he ran,*
*Whene'er he went to pray.*

*A kind and gentle heart he had,*
*To comfort friends and foes;*
*The naked every day he clad,*
*When he put on his clothes.*

*And in that town a dog was found,*
*As many dogs there be,*
*Both mongrel, puppy, whelp and hound,*
*And curs of low degree.*

*This dog and man at first were friends,*
*But when a pique began,*
*The dog to gain some private ends,*
*Went mad and bit the man.*

*Around from all the neighbouring streets*
*The wondring neighbours ran,*
*And swore the dog had lost his wits,*
*To bite so good a man.*

*The wound it seemed both sore and sad*
*To every Christian eye;*
*And while they swore the dog was mad,*
*They swore the man would die.*

*But soon a wonder came to light,*
*That showed the rogues they lied,*
*The man recovered of the bite,*
*The dog it was that died.*

12. Oliver Neville – *Emerods.* We thank Dr. Warin for bringing to our notice an early literary reference to haemorrhoids and their cure. *The Bible. Samuel 1, Chapter 5, verse 1.*

As the original will be on the readers' shelves, this extract is not reproduced here (Ed.)

13. Oliver Neville – Our last word is from *Lord Horder* to his fellow doctors –

Put not your faith in politicians of whatever party, they come and go; but medicine goes on forever. The first is ephemeral, the last is eternal.

